# The genetic basis of the immune response to SARS-CoV-2 infection and vaccination in the Italian municipality of Vo’

**DOI:** 10.3389/fimmu.2026.1718158

**Published:** 2026-03-06

**Authors:** Ettore Zapparoli, Enrico Lavezzo, Hélène Tonnelé, Fabio Simeoni, Jing Guo, Klaudia Walter, Anna Sofia Tascini, Sodbo Sharapov, Rebecca Elyanow, Martina Bado, Giorgia Mazzotti, Dmitry Penkov, Marco J. Morelli, Dejan Lazarevic, Nicola Pirastu, Nicole Soranzo, Ilan R. Kirsch, Andrea Crisanti, Stefano Toppo, Paolo Provero, Giovanni Tonon

**Affiliations:** 1 Center for Omics Sciences, IRCCS San Raffaele Scientific Institute, Milan, Italy; 2 Department of Molecular Medicine, University of Padova, Padova, Italy; 3 Department of Neurosciences “Rita Levi Montalcini”, University of Turin, Turin, Italy; 4 Wellcome Sanger Institute, Cambridge, United Kingdom; 5 Università Vita-Salute San Raffaele, Milan, Italy; 6 Human Technopole, Milan, Italy; 7 Adaptive Biotechnologies, Seattle, WA, United States; 8 Department of Haematology, Cambridge Biomedical Campus, Cambridge, United Kingdom

**Keywords:** COVID-19, HLA, MHC, SARS-CoV-2, T cells

## Abstract

**Introduction:**

It is becoming increasingly evident that SARS-CoV-2 infection is here to stay. Therefore, understanding whether genetic variants may impact the response to the virus or vaccination is crucial. Studies on the genetic determinants of immune responses to SARS-CoV-2 have been limited by the scarcity of genetically homogenous populations and longitudinal designs that assess responses to both infection and vaccination in relation to individual genetic variation.

**Methods:**

Here we performed genotyping and whole-genome sequencing in a well-annotated and intensively followed population from the municipality of Vo’, which has previously provided critical insights into SARS-CoV-2 transmission, infection dynamics and COVID-19 clinical manifestations.

**Results:**

We identified 99 variants within the major histocompatibility complex (MHC) associated with altered T cell response dynamics following infection. These variants clustered into two semi-independent linkage disequilibrium (LD) blocks, respectively tagged by the HLA-A*01:01 allele and by SNP rs1611581. Additionally, when examining the response to vaccination, we identified 617 MHC genetic variants clustering into 27 semi-independent LD blocks that correlated with either increased or decreased TCR responses. We constructed a polygenic risk score (PRS) that comprehensively captures this genetic variation. Finally, structural modelling of selected variants affecting HLA proteins identified specific amino acid residuals most likely to influence interactions with SARS-CoV-2 epitopes, including arginine at position 114, isoleucine at position 97, and alanine at position 152 of the HLA-A molecule.

**Conclusion:**

Together, these findings provide robust evidence that genetic profiles modulate the immune response to SARS-CoV-2 in a longitudinal setting, offering insights that may inform further public health interventions.

## Introduction

The Public Health Emergency of International Concern (PHEIC) caused by SARS-CoV-2 has been declared over by the World Health Organization (WHO) on May 5th 2023. The pandemic is however still ongoing, with new cases and deaths being reported daily. According to the World Health Organization (WHO), as of December 14, 2024, there have been over **7**76 million confirmed cases of COVID-19 worldwide and over 7 million deaths (https://covid19.who.int/). The outcome of the exposure to SARS-CoV-2 is widely variable, ranging from asymptomatic viraemia to severe disease, or even complete resistance to the infection.

As in other infectious diseases (1–3), in the case of SARS-CoV-2, the identification of host genetic factors associated with the various stages of SARS-CoV-2 infection and the clinical manifestations of COVID-19 remains a crucial bottleneck, both from a medical and a public health perspective. An individual’s genetic background may affect all phases of the viral-host interaction, including susceptibility, the ability to mount an appropriate immune response, the variable reaction of the organism, and more broadly the entire spectrum of the clinical response. Despite the extensive literature elicited by the SARS-CoV-2 pandemic, some of these questions have not been properly addressed, due to lack of sound epidemiological and technical evidence.

From the epidemiological perspective, international consortia have collected, merged and analyzed genetic data from a large number of individuals infected with SARS-CoV-2. However, the populations included in these large studies are heterogeneous, potentially weakening the search for polymorphisms associated with patient phenotypes and shared across individuals. This is true in particular for essential genetic variants such as the MHC locus, which is pivotal in eliciting a proper adaptive immune response to viral infections and vaccinations. Additionally, follow up assessment of the same individuals is available only in a handful of studies, and very few studies have explored genetics in non-hospitalized cohorts (4–7). A significant exception is (8), who used longitudinal data in a large UK Biobank cohort to study the genetic determinants of response to vaccination; however their analysis was limited to the antibody response.

Finally, technical issues have hampered a comprehensive assessment of the potential associations between genetics and the response to SARS-CoV-2 infection. Most studies have leveraged genotyping followed by imputations, while very few studies using whole genome sequencing (WGS) have been reported. The availability of WGS data has been strongly advocated (9). To identify genotype-phenotype associations, these studies focused on the more frequently and robustly recorded phenotypes across populations, such as infection severity, which are easy to infer from hospitalization records. Other more specific clinical phenotypes, such as susceptibility towards SARS-CoV-2 infection, are more difficult to obtain, while potentially more relevant for understanding the pathogenesis of the infection and for providing public health guidance. Other relevant non-clinical phenotypes have also been only partially explored. For example, the study of the immune response against SARS-CoV-2 has mostly focused on the B cell response, assayed by the antibody levels, a straightforward measure. However, the T cell response, albeit more difficult to assay, exerts a central role in SARS-CoV-2 infection and provides an index of an effective immune reaction that measures more reliably the ability of the individual to withstand SARS-CoV-2 infection, as well as the response to vaccination (10).

The Vo’ cohort includes about 80% of the population of Vo’, a town in Northern Italy that experienced Italy’s first COVID-19-related death back in February 2020. The cohort has been extensively studied during the COVID-19 pandemic, including multiple follow-ups, for the investigation of infection dynamics, serological response, T cell immunity, response to vaccination, and viral evolution (11–15). Additional information was collected regarding demography and health records, including COVID-19 symptoms, hospitalization, comorbidities and drugs.

The integration of swab testing and serology, together with detailed information on household composition and contact networks, enabled the identification of all the individuals who were infected with SARS-CoV-2 and all those who remained uninfected despite clear exposure to the virus.

**Figure 1:**
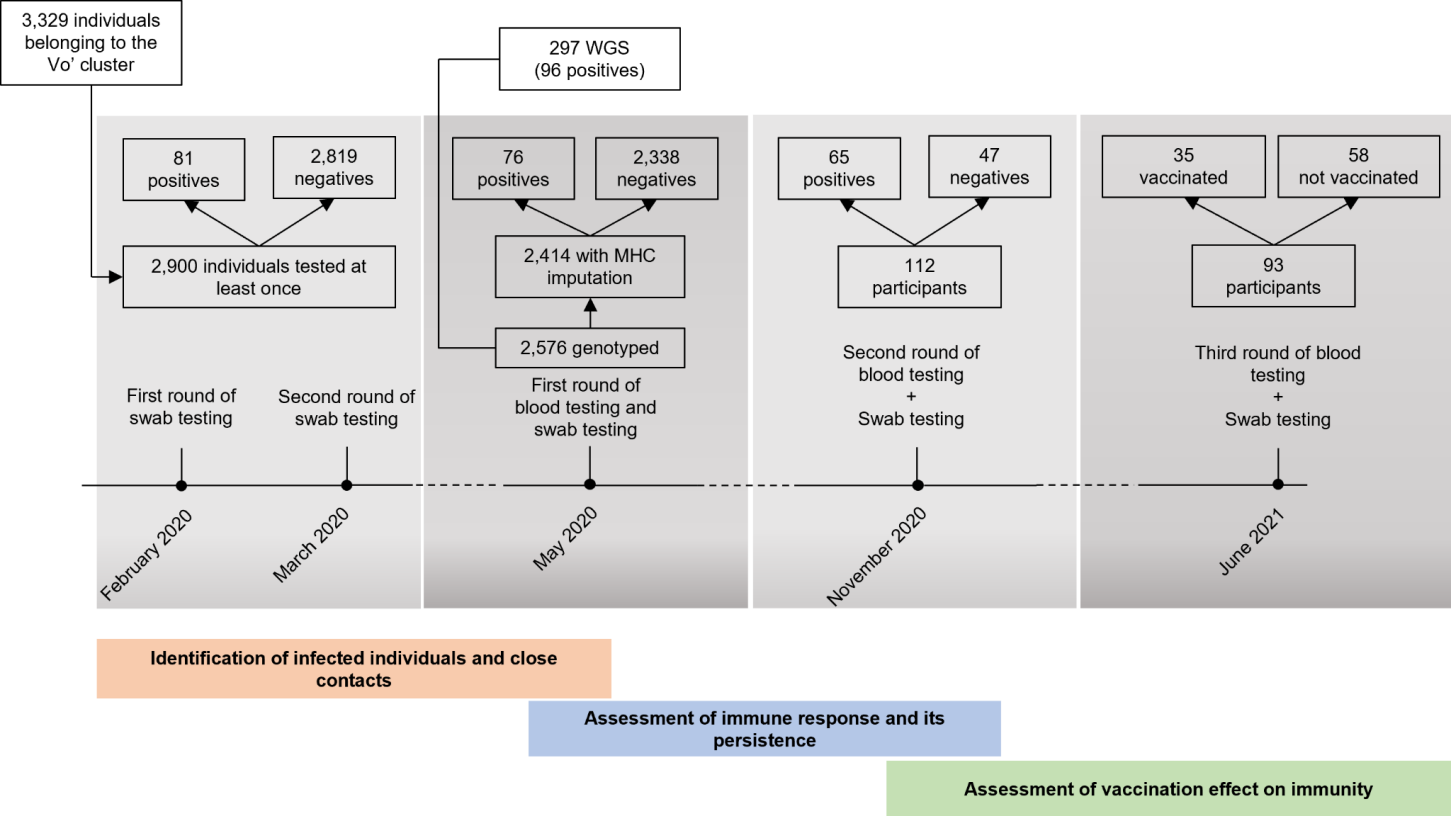
Flow chart illustrating the study design and the number of participants at the different follow-up surveys, divided in positive and negative to SARS-CoV-2 infection. WGS, whole genome sequencing.

In this study, we have genomically profiled the Vo’ population (**Figure 1**; **Table 1**). We have now sequenced 2,576 genomes, leveraging both genotyping and WGS, seeking to define associations between the genetic profile of the MHC locus with T and B cell responses, before and after vaccination. We identified MHC variants associated with the extent of the T cell response both after infection and vaccination. We provide to the scientific, clinical and public health community a carefully and thoroughly annotated dataset, which could be leveraged for discovery, validation of other large yet heterogeneous datasets, and for designing public health strategies.

**Table 1:** Demographic and clinical characteristics of participants at each serosurvey time point.

Characteristic	Category	May 2020 (n=2414)	November 2020 (n=135)	June 2021 (n=115)
Age group, n (%)	0 - 10	116 (4.8)	3 (2.2)	2 (1.7)
	11 – 20	194 (8.0)	5 (3.7)	4 (3.5)
	21 - 30	261 (10.8)	9 (6.7)	8 (7.0)
	31 - 40	269 (11.1)	18 (13.3)	11 (9.6)
	41 – 50	382 (15.8)	15 (11.1)	10 (8.7)
	51 – 60	449 (18.6)	31 (23.0)	28 (24.3)
	61 – 70	351 (14.5)	28 (20.7)	30 (26.1)
	71 - 80	264 (10.9)	24 (17.8)	19 (16.5)
	81 – 90	120 (5.0)	2 (1.5)	3 (2.6)
	91+	8 (0.3)	–	–
Gender, n (%)	Male	1166 (48.3)	64 (47.4)	52 (45.2)
	Female	1248 (51.7)	71 (52.6)	63 (54.8)
BMI category, n (%)	< 18,5	173 (7.2)	4 (3.0)	4 (3.5)
	18.5 – 24.9	1059 (43.9)	57 (42.2)	45 (39.1)
	25.0 – 29.9	779 (32.3)	43 (31.9)	38 (33.0)
	≥ 30.0	271 (11.2)	19 (14.1)	18 (15.7)
	NA	132 (5.5)	12 (8.9)	10 (8.7)
Prior SARS-CoV-2 infection, n (%)	Yes	95 (3.9)	82 (60.7)	70 (60.9)
	No	2319 (96.1)	53 (39.3)	45 (39.1)
Vaccinated, N (%)*	Yes	–	–	49 (42.6)
	No	–	–	66 (57.4)

*Vaccination was not available prior to June 2021 serosurvey.

Values are reported as n (%) unless otherwise indicated.

## Results

### MHC-dependence of TCR dynamics in patients infected by SARS-CoV-2

To ascertain the genetic landscape of the Vo’ population, we genotyped 2,576 individuals using microarrays (**Figure 1**). We also selected 297 individuals, 96 among the positive subjects, 201 among the negative ones but with a strict contact with at least one positive patient, and proceeded with whole genome sequencing (WGS). Preliminary analysis of the genotyping data did not produce associations with phenotypes reaching genome-wide statistical significance, as expected from the relatively small size of the cohort.

Therefore we decided to focus on the MHC region, which orchestrates the immune responses to viruses, modulating the activity of both the B and T cell responses. We hence asked whether any variant within the MHC locus impacted on these immune responses in the subset of individuals infected with SARS-CoV-2. The individuals included in this analysis were infected before May 2020, and assessed at 2 time points, in May and in November 2021. Information on common household, close relatedness, population structure, age, sex, and BMI, were used as covariates as described in the Methods.

We started with the assessment of B cell response, as determined by three different antibody assays (Abbot, Diasorin, and Roche) (13). We found no significant associations between MHC variants within this population and the B cell response.

We conducted a similar analysis probing the T cell response. To this end, we relied on the Adaptive Biotech assay (14). This test provides information on both the breadth and depth of the T cell response (16), assessing respectively the relative number of distinct SARS-CoV-2–associated T cell clonotypes, and a measure of the extent to which clonotypic T cells have expanded. To avoid possible biases of the assay related to HLA genotypes, we limited the analysis to traits defined as differences in TCR breadth/depth between time points measured in the same subject, thus focusing on the dynamics of the T cell response.

**Figure 2:**
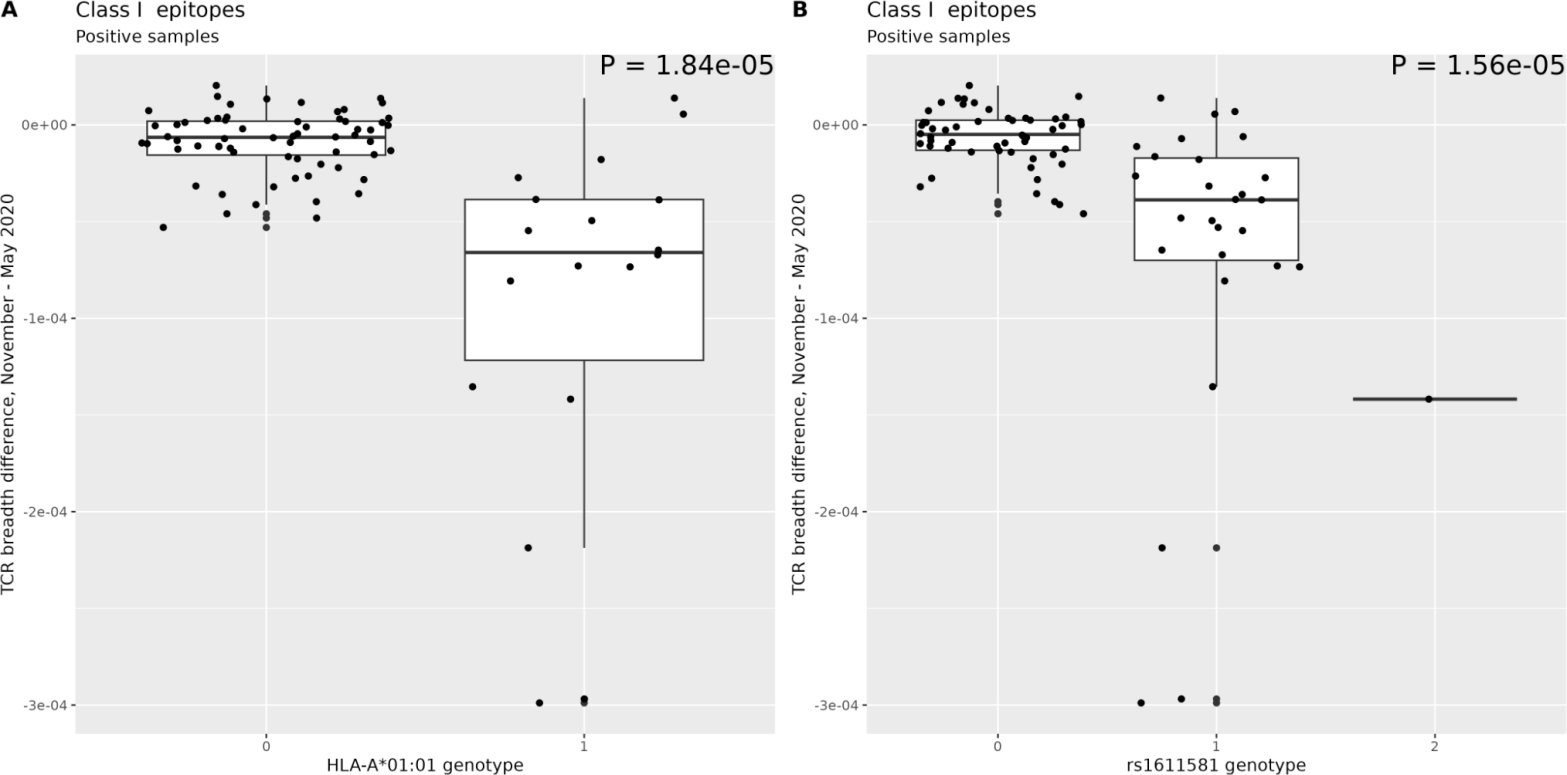
Difference in class I TCR breadth between May and November 2020 for subjects that had been infected prior to May 2020 as a function of the dosage of the HLA-A*01:01 allele **(A)** and of SNP rs1611581 **(B)**. In each figure, the x axis represents the dosage of the variant. The two loci are in moderate LD (R2 = 0.41). Similar results (**Supplementary Figure 1**) are found for Class I TCR depth.

We identified 91 and 99 MHC loci associated with Class I TCR breadth and depth, respectively (**Supplementary Data 1**), with a FDR of less than 5%. Notably, these variants were confirmed in the WGS data (identical genotype in 98.2% of the subject/variant pairs measured with both assays; **Supplementary Data 2**). Most of the significant loci were in linkage disequilibrium (LD; r^2^ > 0.5) with the classic HLA-A*01:01 allele. Specifically, this allele was associated with a markedly faster reduction of Class I TCR breadth (**Figure 2A**) and a similar decrease in depth (**Supplementary Figure 1**). Another SNP, rs1611581, located ~5 Kb upstream of pseudogene HLA-H and in moderate (r^2^ = 0.41) LD with HLA-A*01:01, showed a similar pattern of association with Class I TCR breadth (**Figure 2B**) and Class I depth, although the latter association was not significant at the 5% FDR threshold. This variant has been previously associated by GWAS to testosterone levels (17), a quantitative trait known to affect T-cell immunity (18).

In all, these data suggest that specific HLA alleles and MHC variants are associated with a waning of the T cell response as time passed since the first infection.

### TCR response to vaccination is affected by genetic variants in the MHC locus

We next investigated whether variants within the MHC locus may also impact the B and T cell responses to vaccination. The Vo’ populations included 93 subjects for whom we had TCR traits measured in November 2020 and June 2021, for 91 of which we also had all the covariates needed by the model. Among these individuals, 55 had been infected before May 2020 while 36 had not.

We considered as traits of interest the differences in TCR breadth and depth between these two time points. Since 35 of these 91 subjects had undergone vaccination between the two time points, we implemented a gene x environment (GxE) analysis to determine whether genetic variants in the MHC locus could influence the B and T cell responses to vaccination. The covariates for the genetic analysis were the same as for the previous analysis, and a binary environmental factor was included representing vaccination. Again, no associations were found between the MHC variants and B cell response, assayed with the three SARS-CoV-2 specific tests.

**Figure 3:**
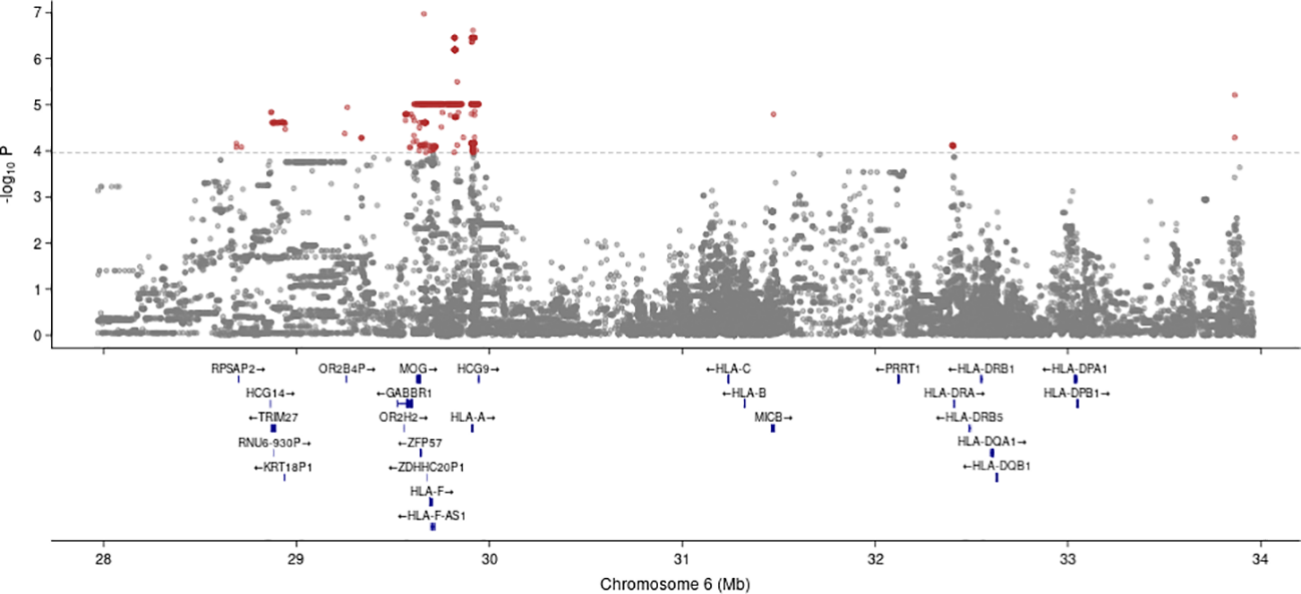
Manhattan plot of the associations between variants in the MHC locus and TCR response to vaccination. The P-values refer to the interaction term between genotype and vaccination status, and the trait is the difference in total TCR breadth between June 2021 and November 2020. Only selected genes are shown (namely the classical HLA genes plus those that are the closest gene to at least one significant variant). The horizontal line corresponds to FDR = 0.05.

Conversely, when we examined the T cell response, the analysis of all subjects revealed a total of 617 significant associations (FDR < 0.05) across all traits considered (TCR breadth and depth, total or restricted to Class I or Class II epitopes), which could be structured into 27 LD blocks (**Supplementary Data 3**). Most of these significant associations (596 variants in 23 LD blocks, **Figure 3**) were found from the analysis of total TCR breadth; three other phenotypes (total TCR depth, breadth limited to class I epitopes, and depth limited to class I epitopes) were associated with one or two blocks only, while no significant association was found for class II depth or breadth. Again, these variants were confirmed in the WGS data (same genotype in 98.5% of the subject/variant pairs measured with both assays - **Supplementary Data 2**). When we limited the analysis to subjects that were respectively positive or negative in May 2020 for SARS-CoV-2 infection, the variants associated with differences in TCR breadth were confirmed in 98% and 99% of cases (nominal P<0.05; 100% concordant sign of effect). For vaccinated subjects, we computed the correlation between the various T cell-associated traits and the time elapsed between vaccination and T cell response assay, which in our sample varied between 8 and 124 days, and could thus in principle affect the measures of T cell response. However, we found no significant association for any trait (all nominal P-values > 0.05), suggesting that the time elapsed from vaccination, within such range, does not significantly affect T cell response.

**Figure 4:**
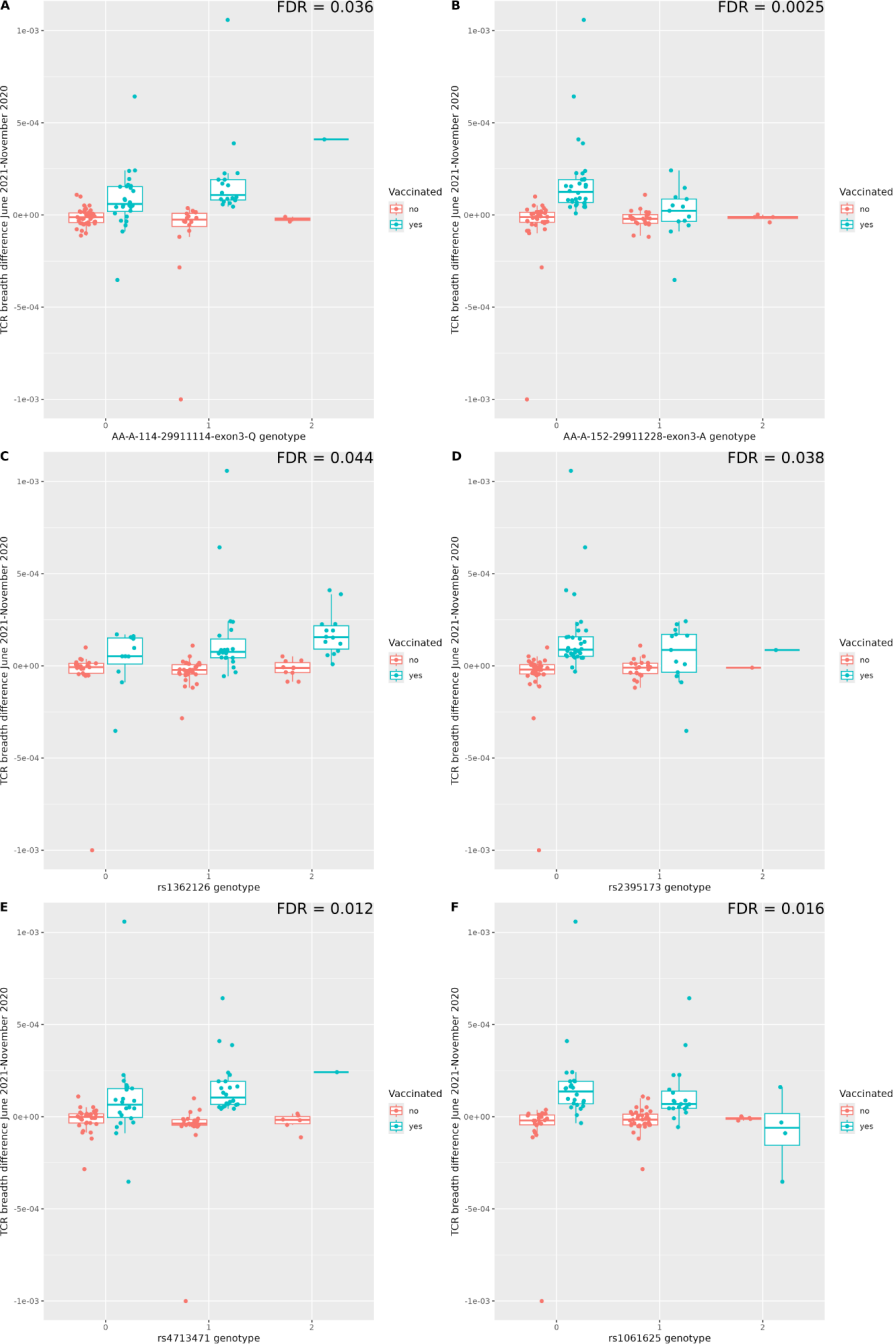
Examples of associations between MHC genotypes and TCR response to COVID vaccination. In each figure, the x axis represents the dosage of the variant. **(A)** Amino acid Q in position 114 of HLA-A increases the TCR response (change in total TCR breadth) to COVID vaccine. **(B)** Amino acid A in position 152 has the opposite effect. **(C, D)** two variants located respectively in the HLA-F and HLA-DRA proximal regulatory regions are associated with higher response. **(E, F)** two variants located respectively in a MICB intron and in the TRIM27 5’ UTR are significantly associated with response in opposite directions. Each variant shown belongs to a different LD block.

Some variants were associated with an increased, while others with a reduced, T cell response. For example, individuals carrying a glutamine at position 114 of the HLA-A protein sequence (positions are referred to protein sequence pdb code 4NQX) had a stronger T cell response to vaccination in terms of TCR clonal breadth (**Figure 4A**), while those carrying alanine at position 152 showed the opposite trend (**Figure 4B**). Other significant LD blocks might point to altered regulation of the HLA genes. For instance, one block tagged by SNP rs1362126 was located in the promoter of non-classical HLA-F gene (**Figure 4C**) while the one tagged by rs2395173 in the HLA-DRA promoter (**Figure 4D**).

Importantly, other signals are located in the wider MHC region, and are likely to affect targets other than the HLA genes. For instance, the SNP rs4713471 is located in an intron of the MICB gene (**Figure 4E**) and rs1061625 in the 5’ UTR of TRIM27 (**Figure 4F**).

To replicate our findings in an independent cohort we obtained TCR response data with HLA typing of 181 healthy controls reported in (19). The genetic data available for this dataset included only HLA typing. Therefore we could only test the associations involving classical HLA types and specific amino acids (whose dosage in each subject we derived by combining HLA typing with the protein sequence of HLA alleles). Thus, nine of the associations we found significant in the Vo’ cohort could be validated in this independent cohort: HLA-A*11:01:01:01, and eight amino acid alleles in HLA-A. Four of them were nominally significant for TCR depth response at 8 weeks in the latter cohort (amino acids I at position 97, A at 152, L at 276, and T at 321, all variants belonging to the same LD block). Four out of five of the remaining associations that could be tested on this cohort showed concordant direction of effect with our results. The mean time elapsed from the first vaccine dose in our cohort was 42 days, thus very similar to the first time point of 8 weeks in the independent cohort. Indeed, none of our significant associations were significant in the independent cohort at 24 weeks after vaccination.

### A polygenic risk score predictive of TCR response to vaccination

**Figure 5:**
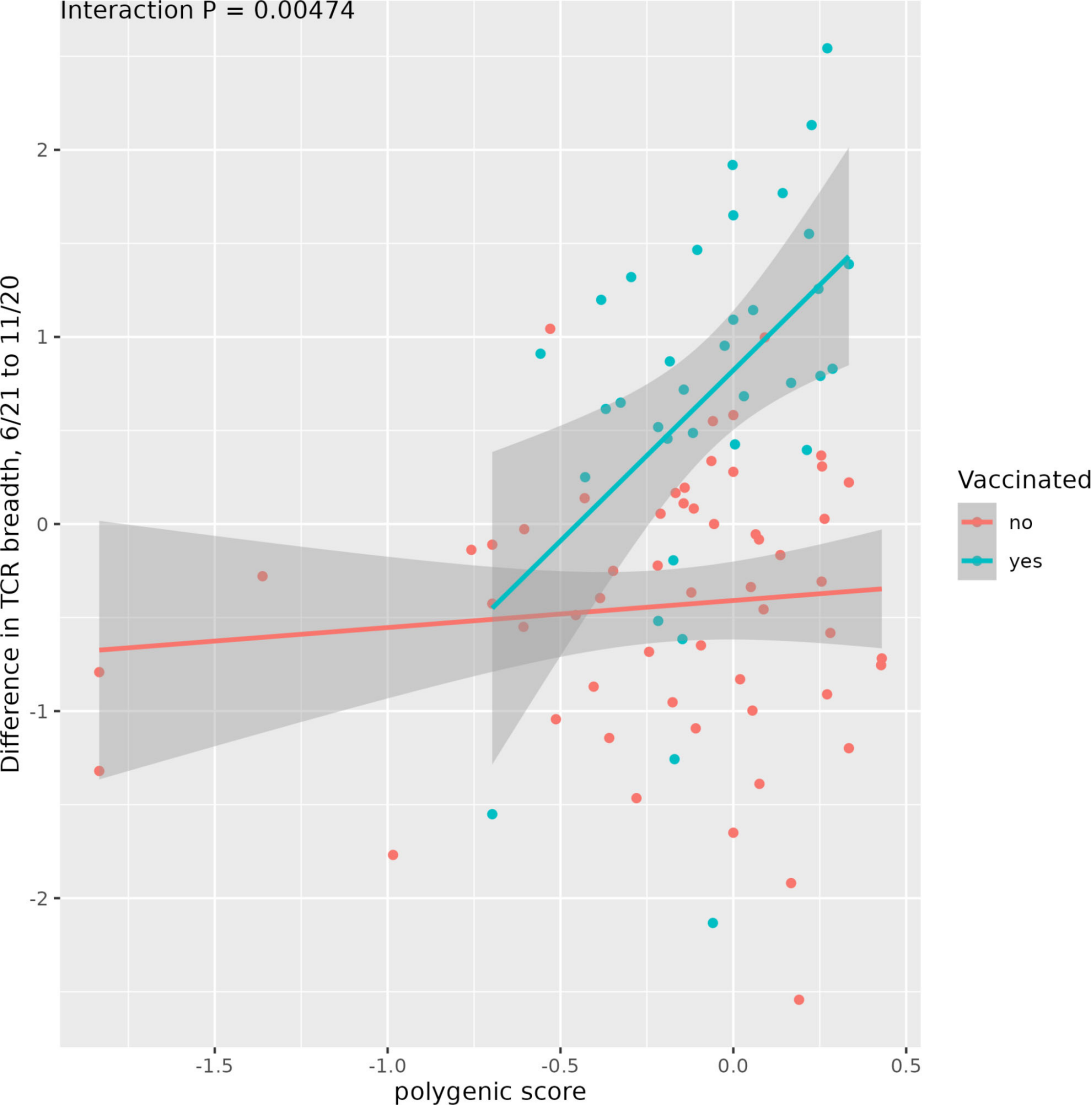
An MHC-restricted polygenic score was built using the variants significantly associated with TCR response to vaccination, and showed excellent predictive performance. The lines represent the best linear regression lines separately for vaccinated and non-vaccinated subjects and their standard error. The polygenic score of each subject was computed through 10-fold cross-validation (Methods).

Having identified 23 semi-independent loci associated with T cell response to the COVID vaccine in terms of total TCR breadth, we reasoned that their contributions could be integrated in a polygenic risk score (PRS) specific to the MHC locus. As described in the Methods, we used a representative variant for each of the 23 loci: the score of a subject was calculated as the mean of the genotypes at these variants, each multiplied by its effect size (namely the coefficient of the GxE term in the linear regression, where the environmental term is the vaccination status). The variants included in the PRS and the corresponding coefficients are found in **Supplementary Data 4**. To validate the predictive power of the PRS, we used 10-fold cross-validation, as detailed in the Methods. We found that the resulting PRS was highly predictive of TCR response (cross-validated P-value 4.7 e-3, P-value on training set 4.3 e-7), as shown in **Figure 5**. While trained on TCR breadth data, this PRS was also predictive of TCR clonal depth, in the expected direction (P = 2.5 -4). The independent cohort used above could not be used to validate the polygenic score due to lack of SNP-level genotyping.

### Epitopes and HLA structure predictions

**Figure 6:**
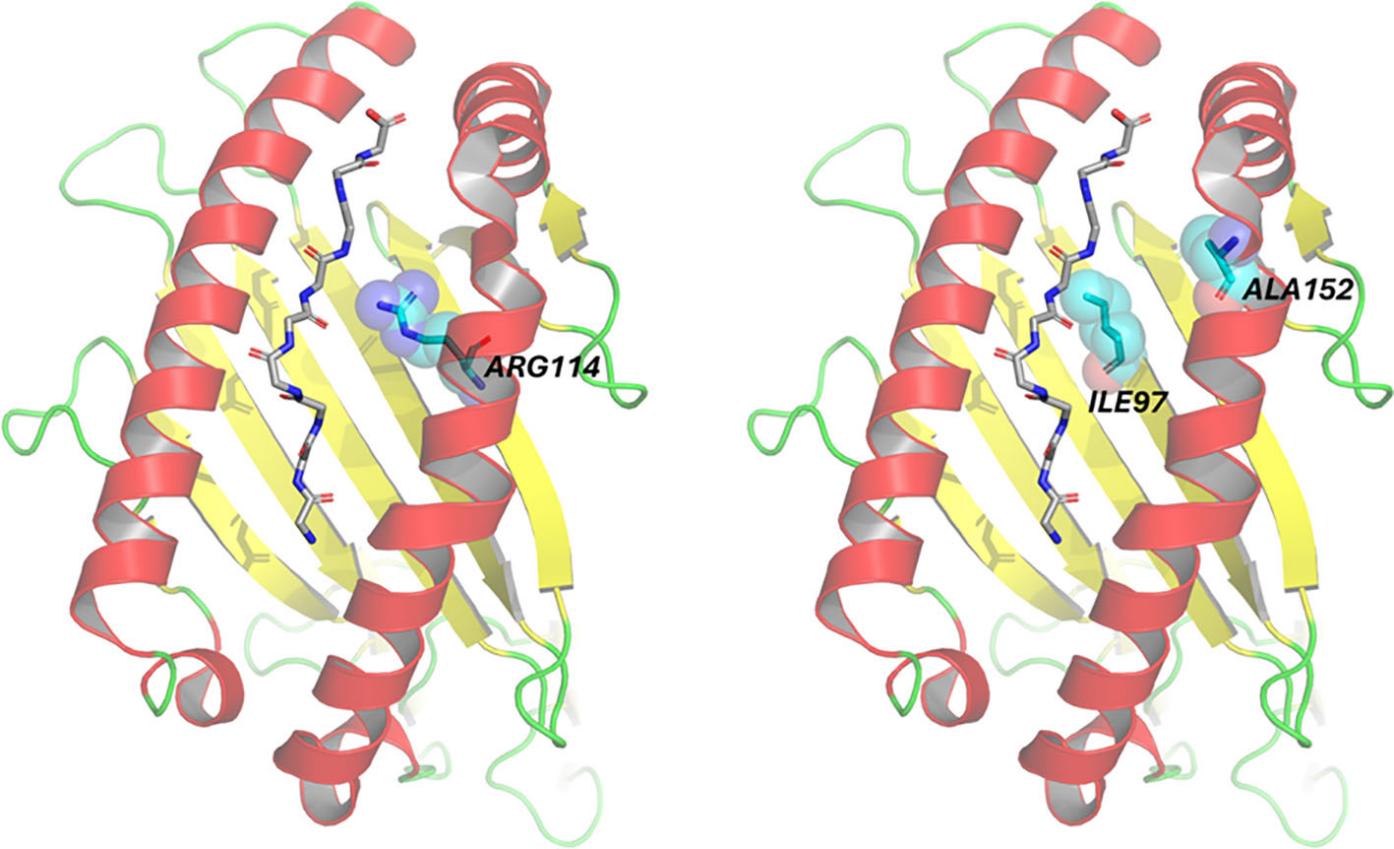
Representation of amino acid positioning within the crystal structure of the HLA-A sequence (PDB: 4NQX). The binding pocket complexed with the epitope is highlighted, along with the variants identified as statistically significant in the genomic analysis. The epitopes are shown in stick format without the side chains of the amino acids whereas the whole protein HLA-A is represented in the cartoon. In the first panel, the variant arginine at position 114 is depicted in ball-and-stick format, corresponding to genomic location chr6:29911114. The second panel also shows two statistically significant variants within the binding pocket isoleucine at position 97 and alanine at position 152 both represented in ball-and-stick format and corresponding to genomic locations chr6:29911063 and chr6:29911228, respectively.

We were then curious to assess whether any of the variants that we found in the MHC locus may potentially impact the interaction between the immune system and the virus. Among the variants significantly associated with the TCR response to vaccination, eight, belonging to two LD blocks, represented amino acid changes in the HLA-A protein. We thus explored the position of these amino acids in the protein structures of HLA deposited in the PDB databank. A representative set of all the available PDB structures was evaluated (see **Supplementary Data 5** in the **Supplementary Methods**), confirming the high conservation of the structural conformation of the HLA (references) and the location of the different epitopes (**Supplementary Figure 1**). To highlight the amino acids of interest, we selected the crystal structure of HLA A*0101 in complex with NP44-S7N, an 9-mer influenza epitope (pdb code 4NQX) as example. The first block included positions 114, 246, 299, and 334 (the coordinates refer to the PDB sequence 4NQX) where glutamine (Q), serine (S), alanine (A), and methionine (M) residues were associated with higher response. In the left image of **Figure 6**, position 114 is an arginine (R) in 4NQX structure as no other sequences in PDB display a glutamine (Q) in that position. The other three residues fall outside the binding groove of the epitope, suggesting a role alternative to the binding of the epitope.

The second block included positions 97, 152, 276, and 321, where specific residues (respectively isoleucine (I), alanine (A), leucine (L), and threonine (T)) were associated with lower response in terms of TCR breadth. Also in this second block, only two amino acids (positions 97 and 152) are highlighted in the structure 4NQX on the **Figure 6**, right, because they are located in the binding cleft of the HLA. Interestingly, the amino acids identified in the cleft of the HLA correspond to the binding pocket “E” (20, 21) and are directly involved in the contact with some residues of the epitope.

## Discussion

In this study we have identified a subset of MHC variants associated in the Vo’ municipality with the T cell response to SARS-CoV-2 infection. We have also determined additional MHC variants impacting on the T cell responses to the vaccination against SARS-CoV-2. We have integrated the MHC variants associated with the response to vaccination into a polygenic risk score predictive of the TCR response. We have finally assessed how some of these variants may impact on the HLA structure and on the interaction between HLA and SARS-CoV-2 epitopes.

Genome-wide analyses, where genotyping has been utilized to impute HLA, have failed to identify strong associations with the disease (22). For example, leveraging on 20,000 cases compared with 2,000,000 controls, the COVID-19 Host Genetics Initiative have identified only one HLA variant, HLA-DRB1*04:01, which confers a modest reduction in risk of critical COVID-19 (OR = 0.80) (23, 24). Possible reasons include the heterogeneity of the underlying population (some studies have even excluded the HLA locus for this reason, (25), the inclusion exclusively of hospitalized patients with severe disease and the lack of longitudinal data. This last feature is critical, as it allows a proper internal control, thus avoiding false positive results. The Vo’ municipality cohort includes almost the entire population of a town in northern Italy that underwent strict lockdown at the height of the SARS-CoV-2 pandemic. This population has been followed along the years, thus providing an almost unique cohort amenable to study a large array of features, including the MHC locus and, specifically, HLA genes, that insofar escaped a thorough assessment. While we have been unable to identify associations between MHC variants and the antibody response to SARS-CoV-2 infection, we found a strong association between MHC variants and both breadth and depth of TCR responses. More specifically, several variants in LD within the HLA-A*01:01 locus were associated with a reduction on the TCR response with time, suggesting that some individuals may be more prone to reinfection due to a waning immune response to SARS-CoV-2 infection. Other studies have reported a role for HLA-A*01:01. Previous analyses have identified an association between HLA-A*01:01 and susceptibility to infection and disease severity (26–29). Our results provide a potential explanation for these phenotypes, suggesting that individuals presenting with this HLA makeup mount a less effective T cell response against the SARS-CoV-2 virus.

The rapid reduction of the immune protection provided by vaccination against infection is one of the central concerns in managing COVID-19 (30–32). However, while the reduction of detectable circulating antibodies has been widely reported, it is emerging more and more clearly that robust T cell responses are maintained through time, and most importantly provide a strong protection against SARS-CoV-2, in particular towards the more severe forms of COVID-19 (6, 10, 33–44). This prominent role of the T cell response has been somehow underestimated (10). Several examples instead point to this central activity of T cells. For example, individuals with either primary or pharmacologic B cell deficiencies show enhanced T cell immunity, after SARS-CoV-2 infection and vaccination (45). These findings in humans are supported also in transgenic mouse models, whereby it has been demonstrated that T cells are essential for clearing the viral infection and resolving the disease (46). The central role exerted by T cells during SARS-CoV-2 infection and vaccination has indeed prompted calls to extend the assessment of the T cell response alongside antibody detection to probe B cell, towards better public health policies and interventions (47). Our findings of MHC variants associated with an increased or reduced T cell response provide key indications for future public health policies, through the stratification of the population based on the MHC genotype. To that end, we built a polygenic risk score that could accommodate the wealth of variants identified in our study, to provide a more robust and comprehensive approach towards the management of these patients. Other efforts, leveraging the UK Biobank dataset and other large data collections have recently been proposed. For example, a study by Fritsche and others has defined a PRS to identify novel associations between pre-existing clinical phenotypes and COVID-19 severity (48). Another study has generated two PRS for susceptibility and severity outcomes based on leading risk-variants from the COVID-19 Host Genetics Initiative (49). None of these PRS have however explored the relationship between MHC genotype and T cell response, nor, to our knowledge, others have reported a similar tool.

The potential structural interactions between the HLA proteins and the SARS-CoV-2 antigens have not been previously explored in detail at the amino-acidic level. We found that in the context of our HLA signature some amino-acidic variants emerged as significant. Of the eight residues found in two distinct LD blocks, interestingly arginine 114 in the first LD block and isoleucine 97 with alanine 152 in the second LD block are placed in the important binding pocket E of the interaction cleft of HLA with the epitopes. This suggests an important role of these amino acids in the HLA variants vs. SARS-CoV-2.

Our study has some limitations: first, the relatively small sample size probably impacted our ability to identify the genetic determinants of the dynamics of B cell response in the MHC locus, in particular to vaccination, that have been found in other works based on much larger cohorts (5, 6). Besides the small sample size, also the use of antibody assays might have limited the resolution of such analysis. Future works with larger cohorts, possibly based on the detailed analysis of the BCR repertoire, will be needed to identify the genetic determinants of B cell response to SARS-CoV-2 infection and vaccination.

Moreover, the design of the assay we used led us to limit the analysis to the dynamic evolution of the T cell response (differences between time points), and prevented us from studying the genetic determinants of the absolute magnitude of the response, which would clearly also be of interest.

Finally, our analysis of the genetic determinants of response to vaccination did not take into account the infection status, which could influence the T cell response through the induction of memory T cells. Separate analyses for positive and negative subjects validated at the nominal level the results obtained from the combined analysis, although the reduced sample size prevented the identification of MHC-wide significant associations. Larger cohorts will be needed to analyze the effects of prior infection on the T cell response to vaccination.

Notwithstanding these limitations, our study has several strengths. While not large, our cohort was genetically and environmentally homogeneous, factors that likely facilitated the identification of significant genetic associations, especially concerning the MHC locus. Data about T cell response are much less readily available than seroconversion data, increasing the significance of our results. Moreover, we could exploit abundant and detailed phenotypic and demographic data, used in the model as covariates, including in particular household annotations, which are obviously of great significance when studying an infectious disease, and are not always available in larger scale studies.

In all, in this manuscript we have identified several variants in the MHC locus that are associated with the dynamics of T cell response after natural infection and after vaccination. Some of these variants are likely to alter the interaction between HLA proteins and the SARS-CoV-2 virus. Together with analogous results that have been published on the B cell response, our data could be used to inform personalized immunization interventions.

## Methods

### Sample collection and processing

Following the first COVID-19 death in Italy in February 2020, the entire population of Vo’, a municipality in the Veneto region, underwent extensive epidemiological and virological investigations. Two cross-sectional surveys were conducted in February and March 2020, involving nasopharyngeal swab collection for RT-qPCR testing to determine the prevalence of SARS-CoV-2 infection (11). Additionally, blood samples were collected at two, nine, and fifteen months after the initial outbreak to assess both serological responses (12, 13) and T cell immunity over time (14). Host genetic factors were investigated through genotyping in all participants and whole-genome sequencing in SARS-CoV-2-positive individuals and their close contacts (**Figure 1**).

SARS-CoV-2 infections were identified using a combination of RT-qPCR results and serological data (13). Demographic and clinical data were collected at the time of sample collection.

### TCR breadth and depth assessment

Peripheral blood samples were subjected to high-throughput T-cell receptor β-chain (TCRβ) sequencing using a multiplex PCR–based assay targeting rearranged V(D)J regions, as previously described (16). Briefly, genomic DNA was amplified using a bias-controlled primer set spanning all functional TCRβ V and J genes, followed by next-generation sequencing. Sequencing reads were processed using a standardized bioinformatics pipeline to identify unique productive TCRβ clonotypes, defined by their V gene, J gene, and CDR3 amino acid sequence. Clonotype frequencies were normalized to the total number of productive templates per sample to enable comparisons across repertoires.

Clonal breadth and depth metrics were derived from a predefined set of SARS-CoV-2–associated public TCRβ clonotypes identified through case–control enrichment analysis. A one-tailed Fisher’s exact test was used to identify 4,469 clonotypes significantly enriched in 784 individuals with real-time PCR–confirmed SARS-CoV-2 infection compared to 2,447 healthy controls collected before 2020. Clonal breadth measures the relative number of distinct SARS-CoV-2–associated T cell clonotypes, and clonal depth measures the extent to which clonotypic T cells have expanded.

### Genotyping

The genotyping array data contained 730,059 variants in 2,576 individuals. After sample QC, 158 samples were removed. In more detail, 67 samples had discordant sex information, 36 samples had call rate <97%, 19 samples were outliers regarding heterozygosity rate (mean +/- 3SD), 14 samples were either duplicates or related individuals with high IBD sharing (PiHat>0.9), 4 samples had duplicated sample ID, 53 samples were likely contaminated, *i.e.* their number of relations was >30 for PiHat>0.1875, and 44 samples were PC-based ethnic outliers. Variants were kept for SNP call rate>99% and HWE p>10^-10^. Variants were removed if there were different missingness rates between cases and controls with p<10^-5^. Autosomal variants were kept and variants on chromosome X. After QC, 532,022 variants were retained for 2,418 individuals with EUR ancestry.

### HLA imputation with TAPAS

We performed variant QC for the genotyped genome-wide variants (m = 730,059) based on SNP call rate >99%, Hardy–Weinberg equilibrium *P* > 10^−10^ and MAF >0.01. Among them, we retrieved 7,415 variants for the region 28-35Mb on chromosome 6 for the individuals after sample QC (above). We further excluded one individual due to the extreme BMI (recorded >100), and only the individuals with both genotype and phenotype data available were kept (n = 2,414). HLA imputation was performed by TAPAS (GRCh37) based on 1000 Genome Project data as the reference panel. In total, 88,279 variants were imputed for that region including 663 HLA classical variants, 1,726 HLA amino acids, 10,715 HLA intragenic SNPs, 162 insertions/deletions, 61,773 SNPs with rsID numbers available and 13,402 others. We further performed QC to the imputed variants based on MAF >0.01 and the estimate of SNP imputation accuracy (dosage R-squared >0.6) and obtained 60,889 variants including 176 HLA classical variants, 1,156 HLA amino acids, 9,974 HLA intragenic SNPs, 129 insertions/deletions, 45,316 SNPs with rsID numbers available and 4,138 others.

### Genotype/phenotype associations

Variants located in the MHC locus and imputed by TAPAS were analyzed for association with TCR depth and breadth. To avoid possible HLA-related bias in the assessment of TCR depth and breadth, only traits defined as differences between time points were analyzed for genetic associations. The association analysis was performed with GENESIS v. 2.32.0 (50) which allows correcting for multiple random effects: In our case kinship and common household were taken into account as random effects, while age, sex, BMI, and the first 10 genomic principal components were included in the model as fixed effects.

All these covariates were used to generate a null model which was then used as input for the association analysis. For the analysis of the dynamic changes in TCR response between November and May 2020 association analysis was performed only for individuals with evidence of exposure to infection. When analyzing differences in TCR traits between June 2021 and November 2020 we considered all subjects irrespective of exposure to infection (as this was not assessed after May 2020), and included in the model a gene/environment (GxE) interaction term, the environmental variable being vaccination status represented as a binary variable. Multiple testing was taken into account with the Benjamini-Hochberg procedure (51) applied to all analyzed traits together, but separately for the purely genetic association (November vs May 2020) and the GxE analysis (June 2021 vs November 2020).

To divide significant results into linkage disequilibrium (LD) blocks we first computed the LD matrix of all the TAPAS-imputed MHC variants found significantly associated with a trait. The LD matrix was then transformed into a network in which an edge joins two variants if their LD R2 is > 0.5. We considered as separate LD blocks the connected components of such network.

### Polygenic risk score

To build a polygenic score for the TCR response to vaccine in terms of TCR total breadth we considered the 23 LD blocks associated with the difference in total TCR breadth between June 2021 and November 2020. For each block we chose the variant with the most significant association P-value. The score of a subject was defined as the mean of the effect sizes associated with these variants, each multiplied by the genotype of the subject. The effect size was defined as the coefficient of the GxE term in the linear regression, where the environmental factor is the vaccination status. The mean was used instead of the customary sum to allow cross-validation, as described below. The score *S_i_* of a subject is thus given by the following formula:


$$S_{i}=\frac{1}{K}\sum_{j=1}^{K}\beta_{j}g_{ij}$$

where K is the number of LD blocks, *β_j_*is the coefficient of the GxE term in the linear regression for the variant tagging the LD block, and *g_ij_*is the genotype (0, 1, or 2) of the same variant in subject *i*.

To assess the performance of the PRS we used 10-fold cross-validation: For each fold, the association between MHC variants and TCR breadth response was evaluated while excluding the subjects in the fold, and a PRS was developed using these associations as described above. This procedure requires, for each fold, the determination of the quasi-independent LD blocks followed by the choice of the most significant variant in each block. Importantly, the number of LD blocks found, and thus the number of variants included in the PRS, will not necessarily be the same for all folds. Thus, to avoid introducing a bias in the PRS due to the variable number of LD blocks, the PRS for each fold was defined as the mean, rather than the sum, of the effect sizes associated with these variants, each multiplied by the genotype of the subject. This PRS was evaluated on the subjects assigned to the fold. By repeating the procedure for all the folds, we obtained for each subject a value of the PRS obtained without using the subject in training the model. The association between this cross-validated PRS and the TCR response was evaluated with a linear model including vaccination status, PRS, and their interaction as independent variables, the interaction P-value representing the significance of the predictive power.

### Validation in an independent cohort

TCR breadth and depth data at baseline and 8 and 24 weeks after vaccination were obtained from the authors of (19). Amino acid dosage at each relevant position was obtained from HLA typing using the reference sequences available from the IMGT/HLA database (https://www.ebi.ac.uk/ipd/imgt/hla/). We considered differences in TCR depth and breadth between 8 weeks and baseline and between 24 weeks and baseline as the quantitative traits to be associated with genetic variants. Associations were evaluated by linear regression after inverse normal transformation of the quantitative traits, with genetic dosage as the regressor of interest and sex and age as covariates. The analysis was limited to individuals classified as of “white” ethnicity.

## Data Availability

Complete summary statistics for the genome-wide association study of antibody and TCR response are deposited at https://zenodo.org/records/18592141. Complete summary statistics for TAPAS-imputed variants are included in the **Supplementary Material**.
