# Supplementary material for: The genetic basis of the immune response to SARS-CoV-2 infection and vaccination in the Italian municipality of Vo’
Source: Front Immunol. 2026 Mar 6;17:1718158. doi: 10.3389/fimmu.2026.1718158 (PMC13002426; doi:10.3389/fimmu.2026.1718158)
Supplement: Supplementary file 1 [file DataSheet1.zip › Supplementary_materials/Supplementary_methods.docx]

## MATERIAL AND METHODS

The reference sequence of HLA (extracted from chr6) has been used to search for highly similar PDB structures using BLAST search vs. PDB databank. A set of 197 highly similar sequences has been obtained and further clustered to remove sequence redundancy at 98% of overall sequence identity using Jalview (Troshin, Procter and Barton, 2011; Troshin *et al.*, 2018). A set of resulting 27 sequences has been obtained and downloaded from PDB. The structures have been superimposed in PyMOL (The PyMOL Molecular Graphics System, Version 3.0 Schrödinger, LLC). The set of sequences is reported in the Table S1 along with their original description.

| **PDB ID** | **Description** |
| --- | --- |
| *6PAG* | HLA class I histocompatibility antigen, Cw-7 alpha chain |
| *8ESH* | HLA-A*02:01 |
| *7SU9* | MHC class I antigen |
| *8SHI* | MHC class I antigen (Fragment) |
| *3BXN* | HLA-B*1402 extracellular domain |
| *6IEX* | MHC class I antigen |
| *7RTD* | HLA class I antigen |
| *7WJ2* | MHC class I protein |
| *7YG3* | MHC class I antigen |
| *8CX4* | MHC class I antigen |
| *1CG9* | PROTEIN (HLA CLASS I HISTOCOMPATIBILITY ANTIGEN, B-35 B* 3501 ALPHA CHAIN) |
| *3SKO* | HLA class I histocompatibility antigen, B-8 alpha chain |
| *4JQX* | HLA class I histocompatibility antigen, B-44 alpha chain |
| *4XXC* | HLA class I histocompatibility antigen, B-18 alpha chain |
| *6MT4* | HLA class I histocompatibility antigen, B-37 alpha chain |
| *7TLT* | HLA class I histocompatibility antigen, A alpha chain |
| *5TXS* | HLA class I histocompatibility antigen, B-15 alpha chain |
| *1IM9* | HLA CLASS I HISTOCOMPATIBILITY ANTIGEN, CW-4 CW*0401 ALPHA CHAIN |
| *6AT5* | HLA class I histocompatibility antigen, B-7 alpha chain |
| *7WZZ* | MHC class I antigen |
| *1EFX* | HLA-CW3 (HEAVY CHAIN) |
| *3W39* | HLA class I histocompatibility antigen, B-52 alpha chain |
| *7MJA* | HLA class I histocompatibility antigen |
| *5IEK* | HLA class I histocompatibility antigen, B-40 alpha chain |
| *6PBH* | HLA class I histocompatibility antigen, A-68 alpha chain |
| *4NQX* | HLA class I histocompatibility antigen, A-1 alpha chain |
| *6J1W* | HLA-A*3001 |
| *6AEE* | HLA class I histocompatibility antigen, alpha chain G |

**Supplementary data 5:** PDB codes of HLA structures used in the structural alignment of Fig. S1

## RESULTS

The whole set of 27 HLA proteins extracted from PDB superimpose perfectly at a RMSD (Root Mean Square Deviation) average of 1.0 Å for the entire length of the 3D structure. Regardless of the epitopes they bind and the side-chains of the amino acids involved in the interaction pocket (see Figure S1) the 3D topology of the backbone of these structures is conserved. The epitopes are highlighted in sticks in the figure and show an extended conformation docked in the binding pocked made of two flanking alpha helices and a beta sheet shown in cartoon.


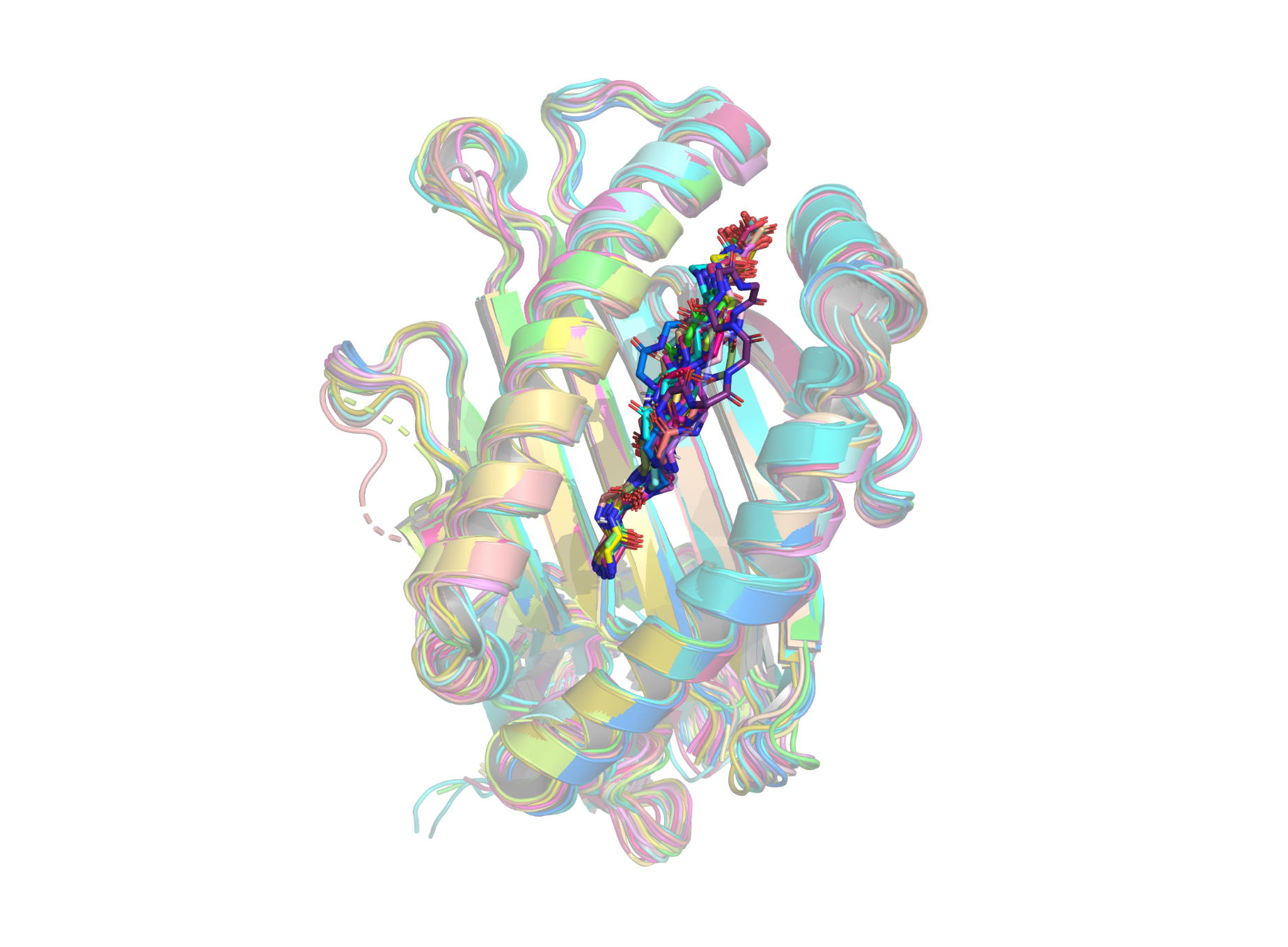


**Fig. S1**: Structural superposition of HLA structures reported in Table S1

## REFERENCES

Troshin, P.V. *et al.* (2018) ‘JABAWS 2.2 distributed web services for Bioinformatics: protein disorder, conservation and RNA secondary structure’, *Bioinformatics*, 34(11), pp. 1939–1940. Available at: https://doi.org/10.1093/bioinformatics/bty045.

Troshin, P.V., Procter, J.B. and Barton, G.J. (2011) ‘Java bioinformatics analysis web services for multiple sequence alignment—JABAWS:MSA’, *Bioinformatics*, 27(14), pp. 2001–2002. Available at: https://doi.org/10.1093/bioinformatics/btr304.
