# Supplementary material for: The genetic basis of the immune response to SARS-CoV-2 infection and vaccination in the Italian municipality of Vo’
Source: Front Immunol. 2026 Mar 6;17:1718158. doi: 10.3389/fimmu.2026.1718158 (PMC13002426; doi:10.3389/fimmu.2026.1718158)
Supplement: Supplementary file 1 [file DataSheet1.zip › Supplementary_materials/Suppl Fig 1.pdf]

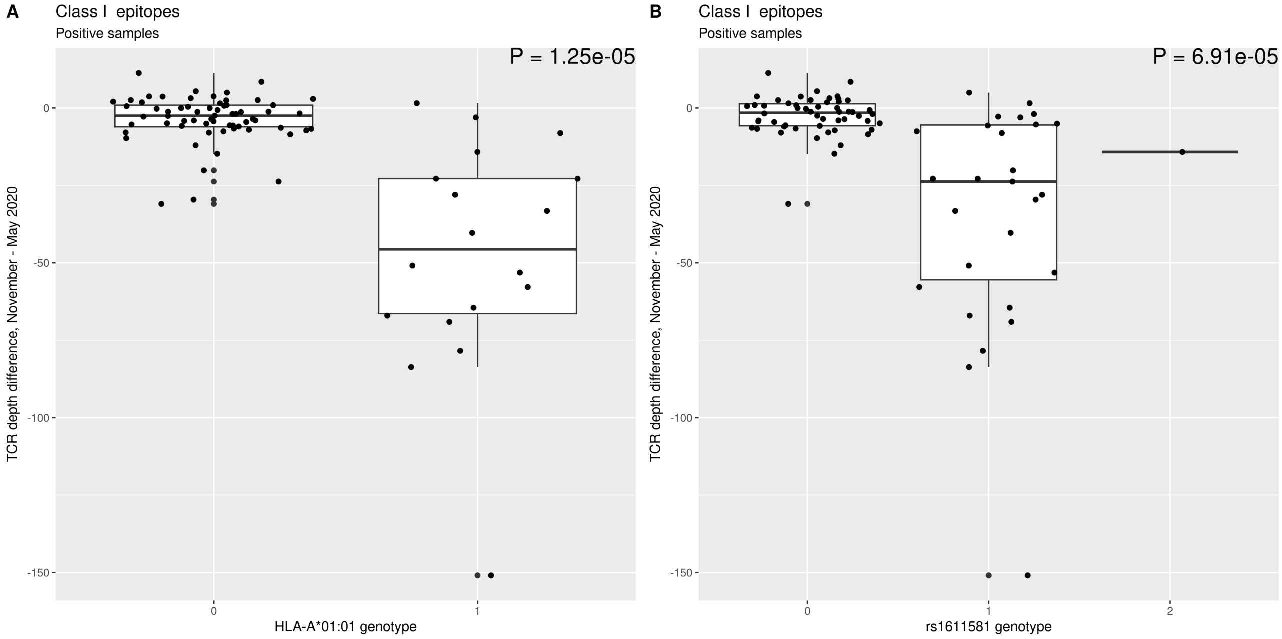

Suppl Fig 1. Difference in class I TCR depth between May and November 2020 for subjects that had been infected prior to May 2020 as a function of the dosage of the HLA-A*01:01 allele and of SNP rs1611581.
